# Digital diagnosis and treatment of mandibular condylar fractures based on Extensible Neuro imaging Archive Toolkit (XNAT)

**DOI:** 10.1371/journal.pone.0192831

**Published:** 2018-02-12

**Authors:** ZhongWei Zhou, Zhang’ao Li, Jiayin Ren, Mingyun He, Yongqing Huang, WeiDong Tian, Wei Tang

**Affiliations:** 1 Department of Oral and Maxillofacial Surgery, General Hospital of Ningxia Medical University, Yinchuan,Ningxia, P.R. China; 2 Department of Oral and Maxillofacial Surgery, West China College of Stomatology, Sichuan University, Chengdu,Sichuan, P.R. China; 3 College of information software engineering, University of Electronic Science and Technology of China, Chengdu, Sichuan, P. R. China; 4 State Key Laboratory of Oral Disease, Sichuan University, Chengdu, Sichuan, P.R. China; Augusta University, UNITED STATES

## Abstract

**Objectives:**

The treatment of condylar fractures has long been controversial. In this paper, we established a database for accurate measurement, storage, management and analysis of patients’ data, in order to help determine the best treatment plan.

**Methods:**

First of all, the diagnosis and treatment database was established based on XNAT, including 339 cases of condylar fractures and their related information. Then image segmentation, registration and three-dimensional (3D) measurement were used to measure and analyze the condyle shapes. Statistical analysis was used to analyze the anatomical structure changes of condyle and the surrounding tissues at different stages before and after treatment. The processes of condylar fracture reestablishment at different stages were also dynamically monitored. Finally, based on all these information, the digital diagnosis and treatment plans for condylar fractures were developed.

**Results:**

For the patients less than 18 years old with no significant dislocation, surgical treatment and conservative treatment were equally effective for intracapsular fracture, and had no significant difference for neck and basal fractures. For patients above 18 years old, there was no significant difference between the two treatment methods for intracapsular fractures; but for condylar neck and basal fractures, surgical treatment was better than conservative treatment. When condylar fracture shift angle was greater than 11 degrees, and mandibular ramus height reduction was greater than 4mm, the patients felt the strongest pain, and their mouths opening was severely restricted. There were 170 surgical cases with condylar fracture shift angel greater than 11 degrees, and 118 of them (69.4%) had good prognosis, 52 of them (30.6%) had complications such as limited mouth opening. There were 173 surgical cases with mandibular ramus height reduction more than 4mm, and 112 of them (64.7%) had good prognosis, 61 of them (35.3%) had complications such as limited mouth opening.

**Conclusions:**

The establishment of XNAT condylar fracture database is helpful for establishing a digital diagnosis and treatment workflow for mandibular condylar fractures, providing new theoretical foundation and application basis for diagnosis and treatment of condylar fractures.

## Introduction

Condylar fracture is the most frequent fracture of mandibular fractures, which often causes pain, swelling, fractured end dislocation, occlusal disorder, etc[1, 2]. Specially, it has potential risk for the development of temporomandibular joint and children's face[3]. Whether to use conservative treatment or surgery to treat condylar fractures has long been controversial[4]. We think that the selection of the treatment plan should base on large amounts of data and accurate measurements. And the scientific prevention and clinical research require a comprehensive database to manage and complete patients’ clinical information. Furthermore, the database can be used as the basis to establish the standardized evaluation system for the prognosis of condylar fractures.

## Materials and methods

### The establishment of condylar fracture database

This study established the condylar fracture database based on XNAT[5]. The data storage is in various forms, including the patient's electronic medical records, 2D photos and 3D face scan data, imaging data, surgical video and so on. It also includes image registration data before and after operation, 3D measurement, stress analysis, computer assisted design and manufacture information, etc. The data processing formats include TXT, JPG, DICOM, STL, OBJ, and WRL. Also, the system integrates the digital soft wares such as (version 4.5 http://www.slicer.org/)、ProPlan CMF、Image J (version 1.49 http://rsb.info.nih.gov/ij/)、Gimias (version 1.6 http://www.gimias.org/)、InVesalius (version 3.0.0 Beta5: http://svn.softwarepublico.gov.br/trac/invesalius) Geomagic studio (Raindrop Geomagic Studio 2013®, Raindrop Geomagic, Inc., NC,USA). Therefore, the system can not only import the data, but also can digitalize the data, such as performing data segmentation, measurement and design.

The study followed procedures in accordance with the 1975 Declaration of Helsinki, as revised in 2000 and was approved by the Ethics Committee of Sichuan University. Full informed consent was obtained from all patients. For the minors, informed consent was obtained from their parents or guardians.

**Fig 1:**
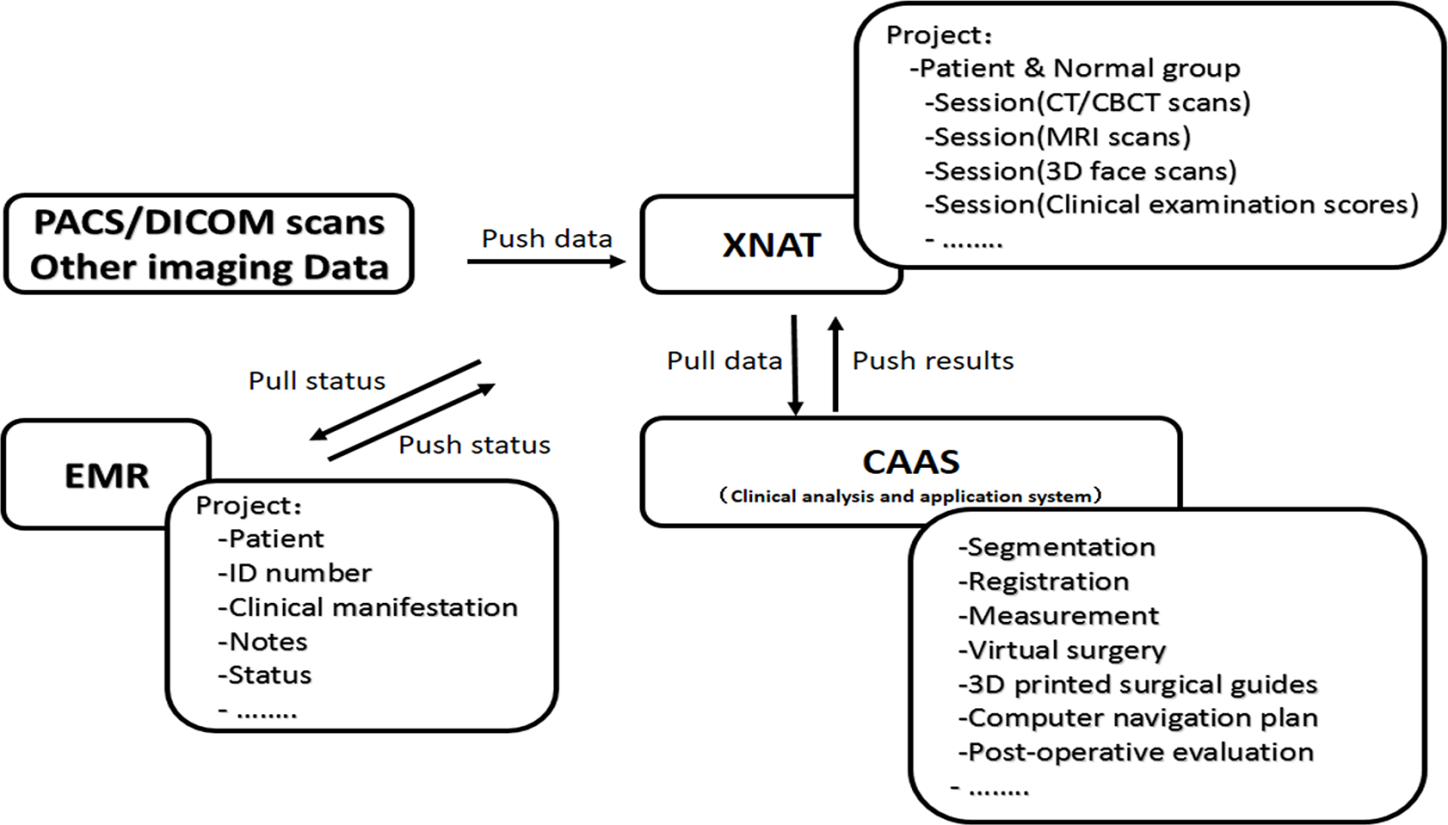
Overview of the system. Black arrows indicate the directions of the data flow.

The server in research center is an enterprise-class 64-bit coordinator server. The system consists of three resource servers designed for different functions (Fig 1):

1. XNAT: receive and store the image data obtained by PACS and other systems, the data type is mainly DICOM;
2. EMR: store the patient-related clinical text data;
3. CAAS: process and analyze data; it integrates a variety of soft wares for data segmentation, registration, measurement and surgical planning;

The data transfer between the three databases uses Python programming language. The raw image data obtained from the multi-source image acquisition device is transferred to the XNAT server through the open source DCM4CHE tool. The data stored on the XNAT server is retrieved using the XNAT software package through python script. Then the data is processed and analyzed on CAAS through a series of standard and customized software packages, and then the results are returned to XANT server.

### The clinical retrospective study of condylar fractures based on XNAT database

We did a retrospective study on the 339 cases included in XNAT condylar fracture database (West China Hospital of Stomatology, traumatic and plastic surgery department, 2011.12–2014.12), and analyzed their age, sex, etiology and fracture types.

### The establishment of digital workflow for evaluation, diagnosis and treatment of condylar fractures

#### The applications of image segmentation, registration and 3D measurement in condylar fractures

Accurate measurement results are helpful for the treatment selection of condylar fractures. The traditional 2D measurement methods set many standards for treatment and analysis. Due to the limitations of 2D, we can only study the tissue changes on cross-section planes. Thereby the images are overlapped along the vertical axis, so the distance and angle measurements are not based on 3D structure, leading to some information loss [6, 7].

In this study, we applied image segmentation and image registration techniques to the treatment of condylar fractures, and quantitatively analyzed the condylar fractures. 53 patients with complete CT data before and after treatment were included in the database. The follow-up period was between 3 months to 3 years, an average of 18 months.

**Fig 2:**
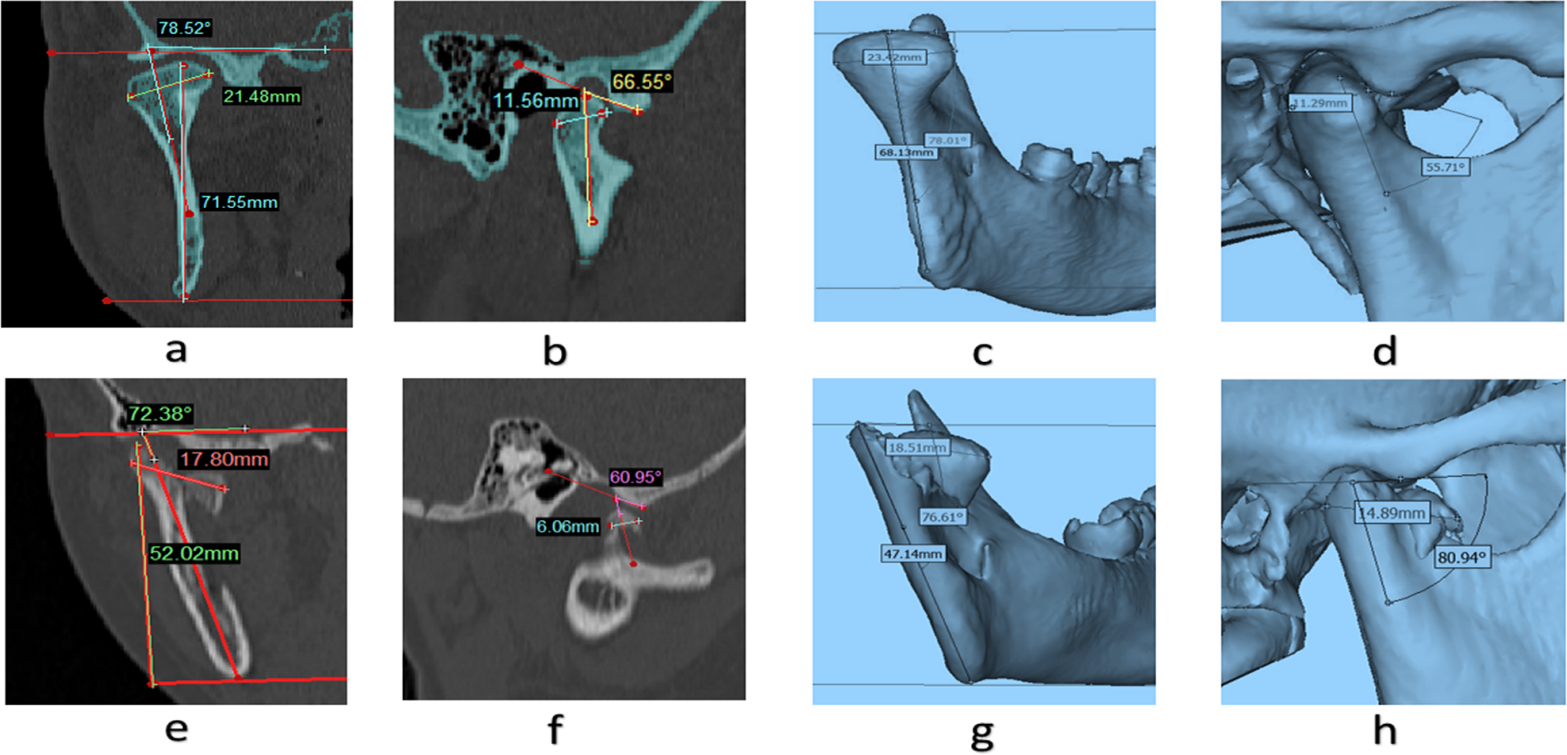
Measurement index (traditional 2D and 3D) in patient and normal case. Condylar Angle in coronal view: Normal case 78.52(a), 78.01(c) respectively; Patient 72.38(e),76.61(g) respectively; Condylar diameter in coronal view: Normal case 21.48(a),23.42(c) respectively; Patient 17.80(e),18.51(g) respectively; Ramus height in coronal view:Normal case 71.55(a), 68.13 (c) respectively; Patient 52.02 (e), 47.14 (g) respectively; Condylar Angle in right sagittal view: Normal case: 66.55(b), 55.71(d) respectively; Patient 60.95 (f), 80.94 (h) respectively; Condylar diameter in right sagittal view: Normal case: 11.56(b), 11.29(d) respectively; Patient 6.06 (f), 14.89 (h) respectively.

Devices used in study:

1. MSCT:16-slice CT scanner (Philips MX16 EVO CT, Holland, 120 kV, 7700 mAS, pixel size: 0.48mm, increment: 0.5 mm, field of view: 250mm, slice thickness: 1 mm, matrix: 512×512 pixels, gantry tilt: 0^o^)
2. Computer: We used a desktop computer Intel Core i7-4770 CPU @ 3.40 GHz, 32.0 GB main memory; Intel, Fort Worth, TX) with the Windows 7 operating system (Microsoft, Redmond, WA).
3. Analysis and measurement software Proplan CMF (Materialise’s Interactive Medical Image Control System, Belgium, Germany)
4. Measurement index (traditional 2D and 3D), shown in Fig 2

2D measurement: the DICOM data in XNAT database was resliced, correcting the deviated axis caused by skewed head position, in order to ensure the accuracy of 2D measurement;

3D measurement: The Proplan CMF software platform in CAAS was used to do image segmentation, which used threshold to segment (HounsField:bone for adult: 226~3071, bone for child: 226~3071);Then the 3D model was reconstructed, measured and analyzed.

**Fig 3:**
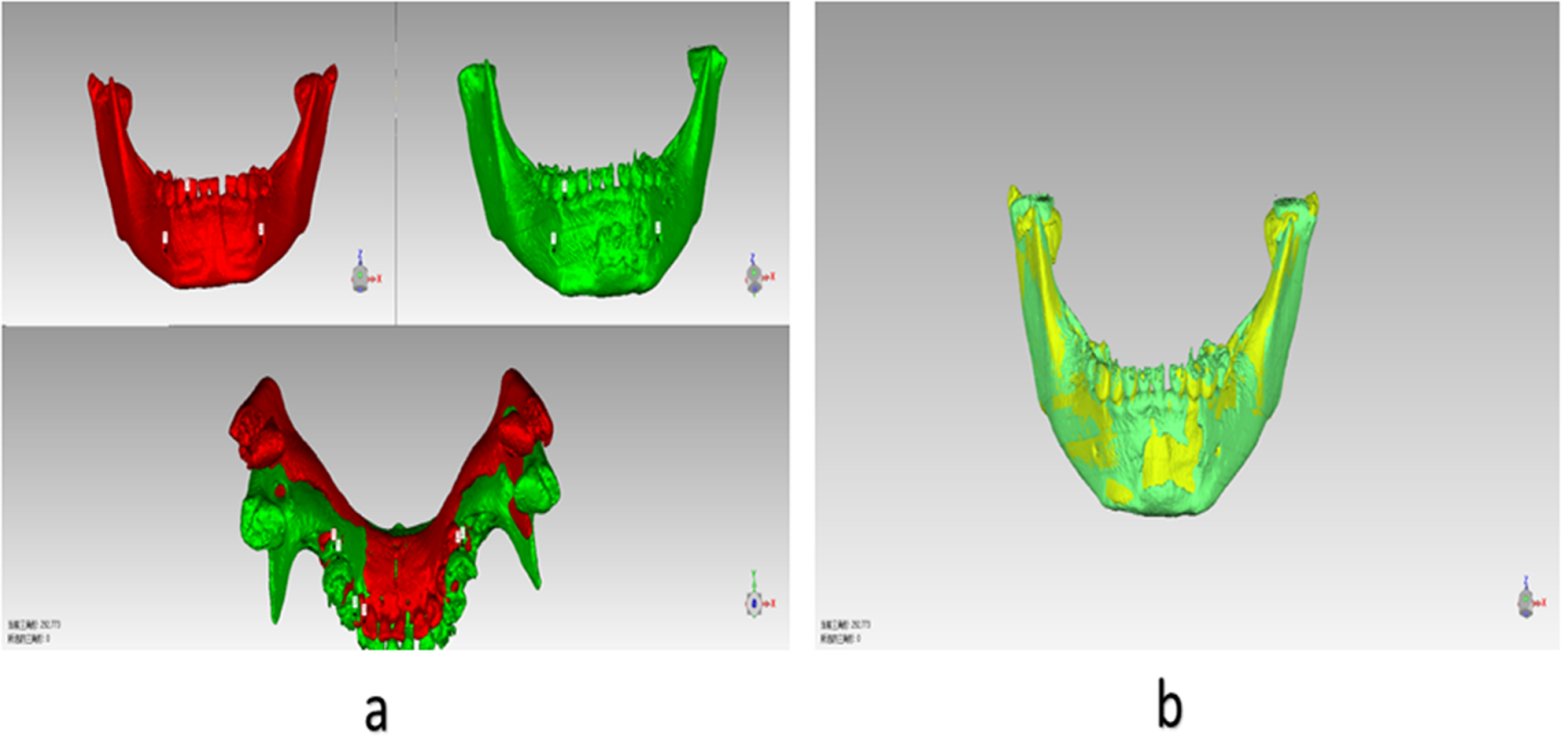
3D model registration process of one case with condylar fracture received conservative treatment before and after operation (a) Procrustes registration system using 3 anthropometric landmarks; (b) Global registration based on the modified ICP algorithm.

**Fig 4:**
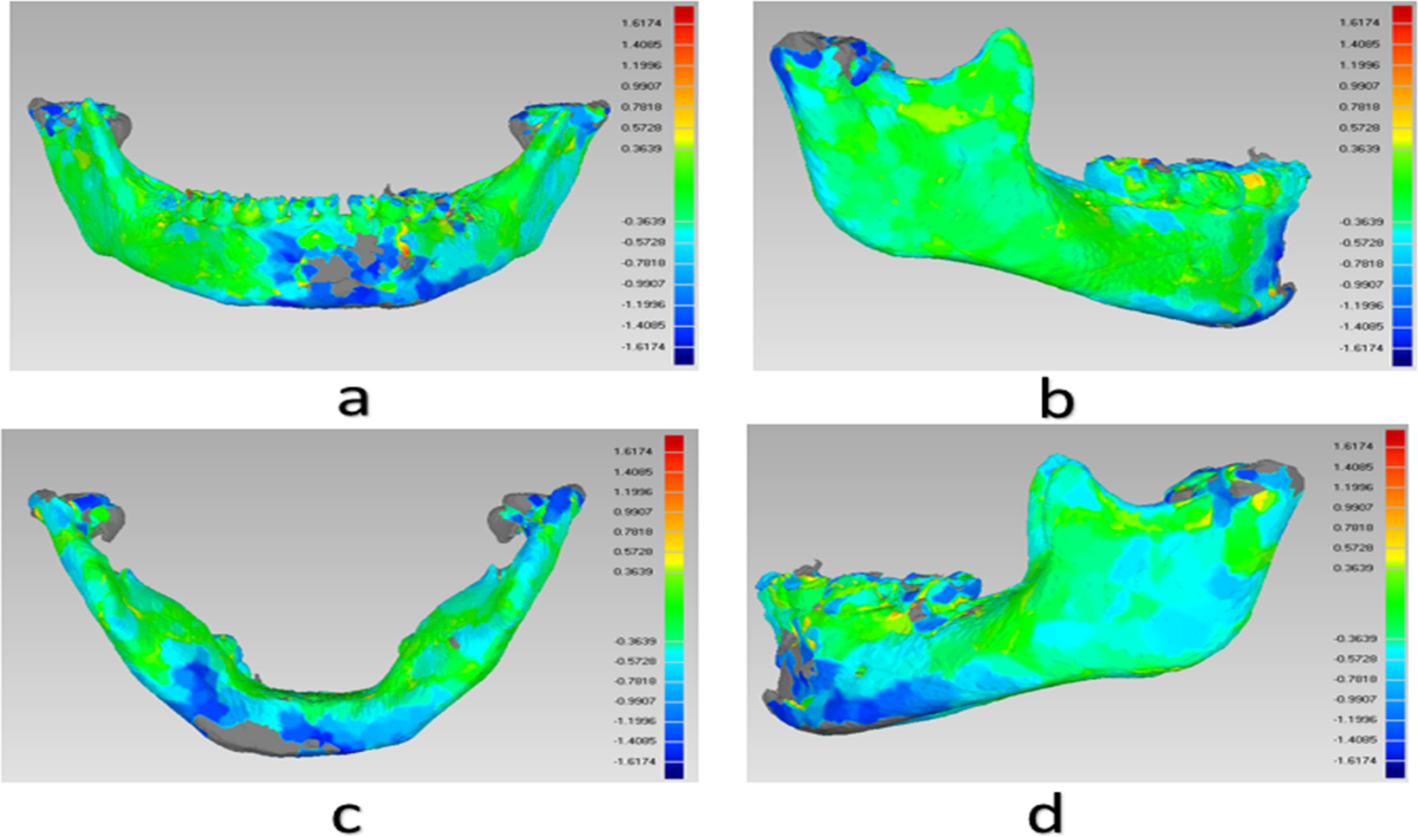
Images of a color-coded surface mismatch error map for one patient received conservative treatment before and after operation: (a) front view, chin fracture after operation; (b) right lateral position, There was almost no changes in mandibular body and angle, suggesting higher fitting degree; (c) bottom view, bilateral condyle changes can be found in this position; (d) left lateral position.

5. 3D data registration analysis before and after treatment: the 3D model produced before and after surgery was exported as STL model, and then imported into Geomagic studio. We then used our improved semi-automatic image registration algorithm to initiate image registration analysis (Fig 3), and exported the chromatograms for evaluation (Fig 4).

Statistical analysis was performed by SPSS 11.5 software to evaluate the difference was considered statistically significant.

#### 180 cases with unilateral condyle fractures were analyzed in this study

**Fig 5:**
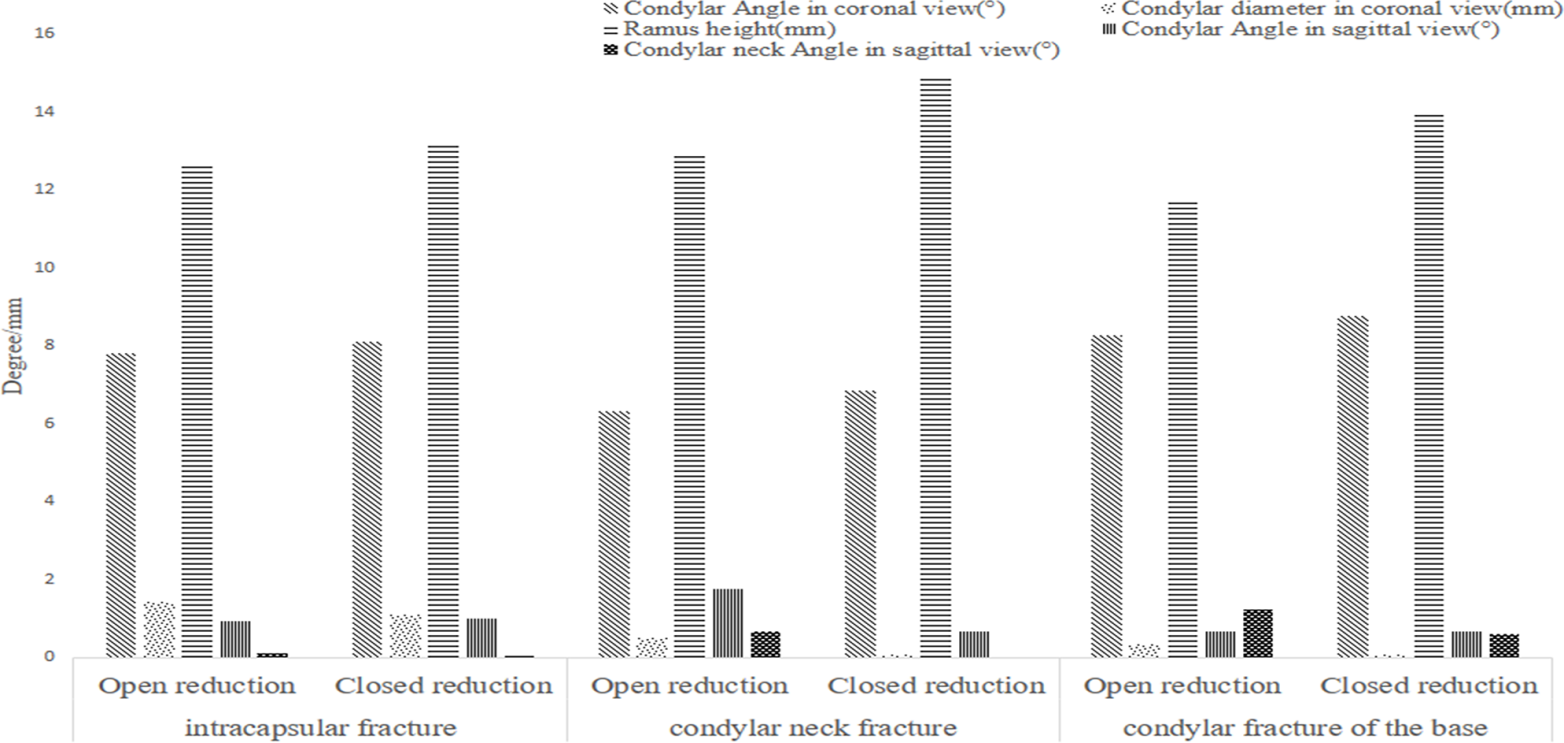
Difference of surgical and conservative treatment data for the patients less than 18 years old.

**Fig 6:**
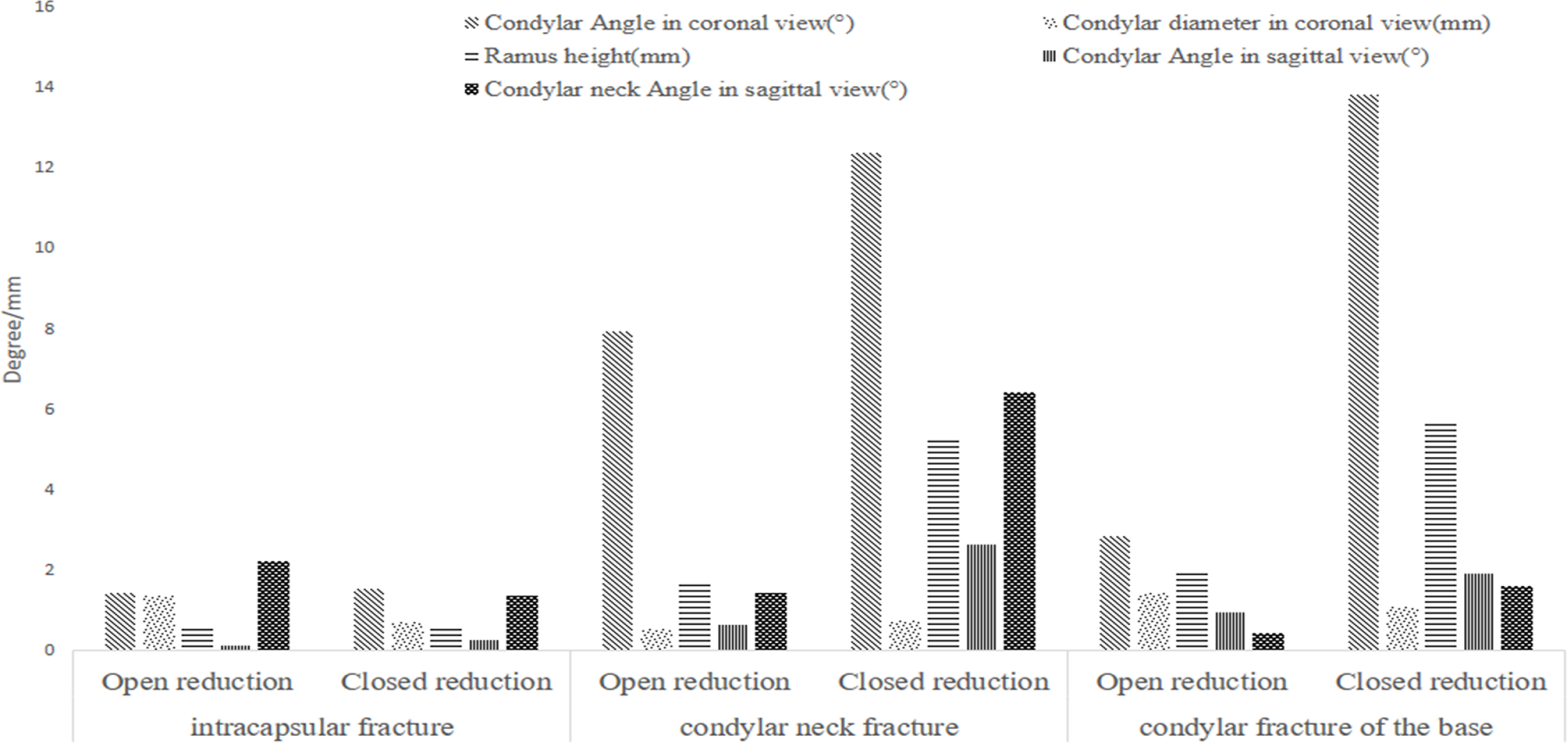
Difference of surgical and conservative treatment data for patients above 18 years old.

We observed the condylar shapes and positions via CT images, and measured the condylar shift angle, the mandibular ramus height reduction, and the anteroposterior diameters of condyle. We also compared these data with the data from patients’ healthy sides, and did quantitative measurement and comparative analysis. The results are shown in Figs 5 and 6.

For the condylar fracture treatment selection in clinic, there are two commonly used evaluation indexes:

Subjective index: PI-NRS pain index[8] (Pain intensity is frequently measured on an 11-point pain intensity numerical rating scale (PI-NRS), where 0 = no pain and 10 = worst possible pain, represents very severe pain that affects life)

Objective index: the maximum opening degree, the distance between the upper and lower central incisor (mm) with maximum mouth opening

## Results

### The establishment of condylar fracture database

The XNAT-based condylar fracture database, which was established by the research center, is stable and feasible. It can conveniently and quickly store the target patient’s data, efficiently inquire the target case, and input the patient data to conduct digital analysis.

### The clinical retrospective study of condylar fractures based on XNAT database

The results showed that: male patients were more than female patients; 18–40 years old was the major age group; traffic factors were the main cause of fractures; condylar neck fracture accounted for the most; and chin injury were frequently involved in combined fractures.

**Fig 7:**
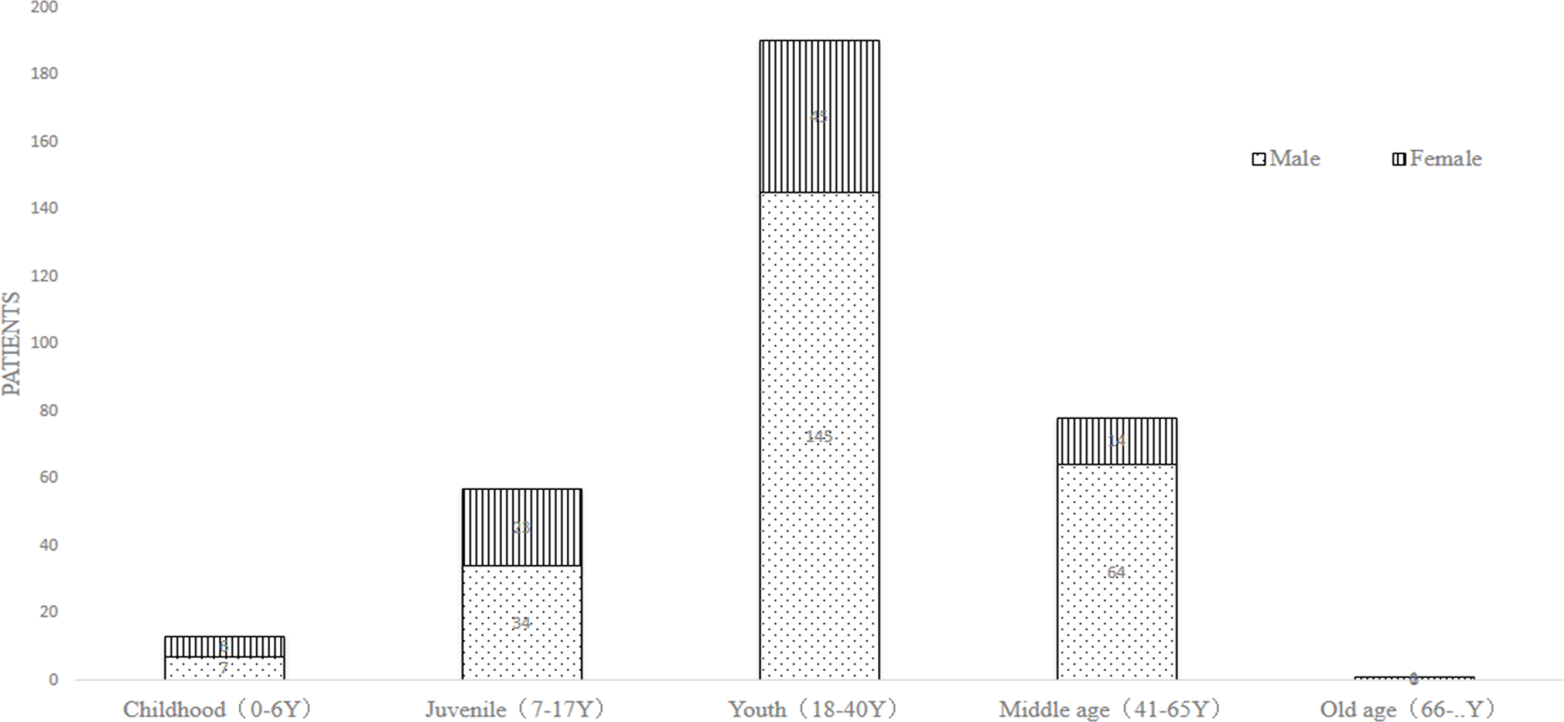
Statistical analysis of condylar mandible fractures in different ages.

The general data analysis showed that: the incidence of males was much higher than females, where males accounted for 74% and females accounted for 26%. The sex ratio of male to female is 2.85: 1 (Fig 7). Condylar fractures were most commonly occurring to young people (18–40 years old), of which the number was 190, accounting for 56%. The gender difference is possibly due to men having more social activities than women in china.

**Fig 8:**
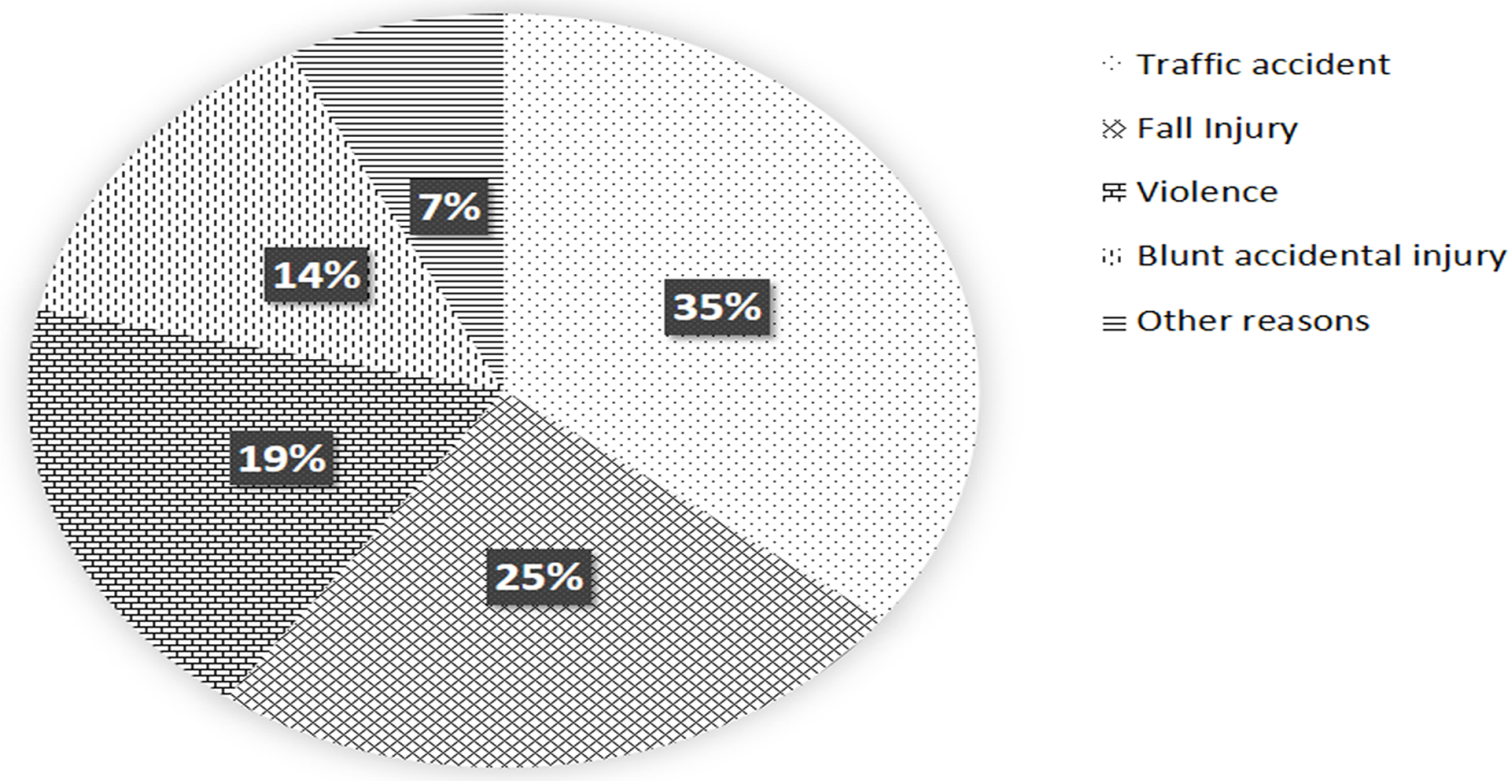
Statistical analysis of etiological factors for condylar mandible fractures.

According to Fig 8, the causing factors of condylar fractures were various, but mainly traffic accidents and fall injury, which accounted for 60% of all cases. This phenomenon is similar to what happens in the developed countries, and is likely due to the continuous growth of motor vehicles in China.

**Fig 9:**
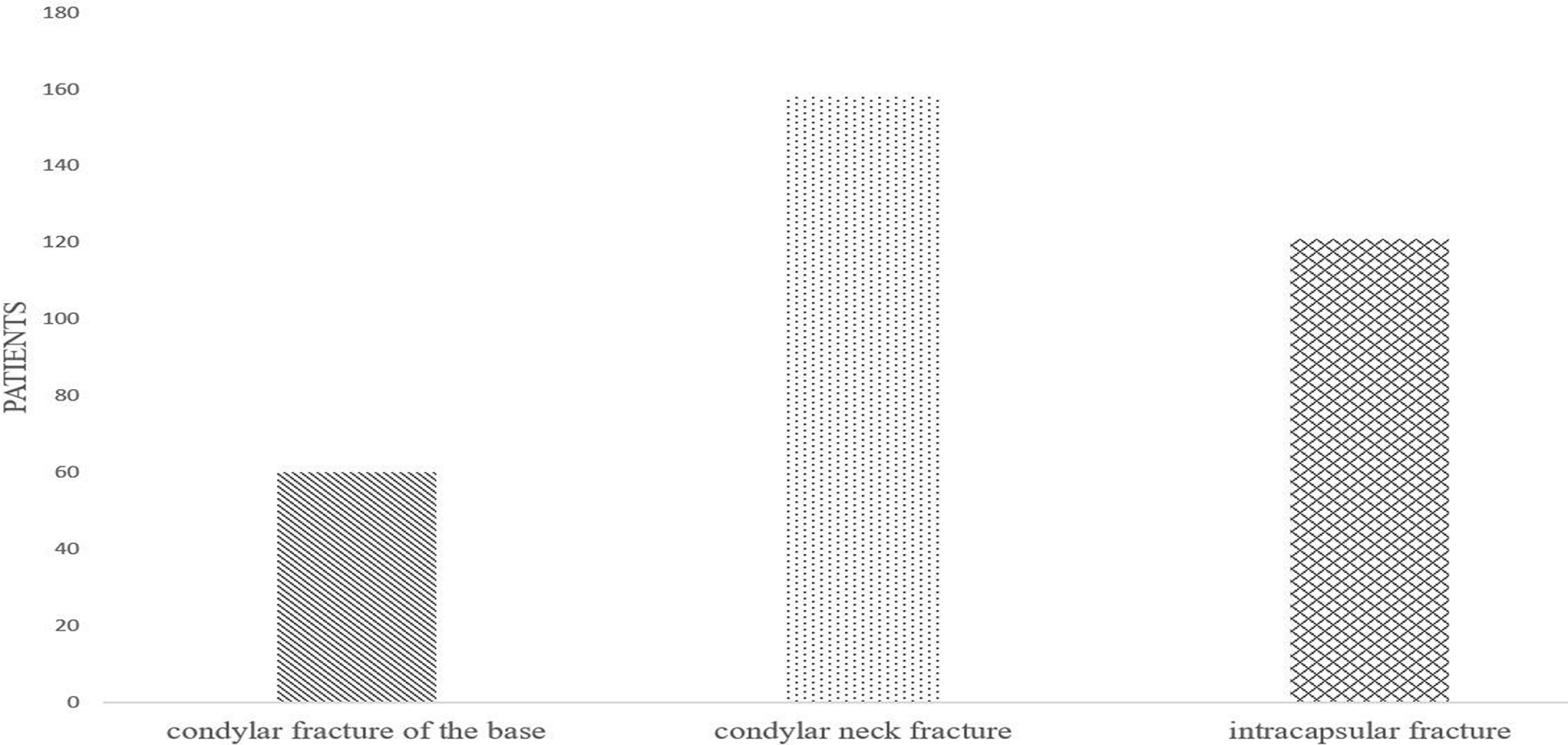
Statistical analysis of etiological factors for condylar mandible fractures.

According to Fig 9, neck fracture is the major type of condylar fractures, accounting for 47% of all cases, while basal fractures and intracapsular fractures accounted for 18% and 35%, respectively. The number of patients with neck fractures was 2.6 and 1.3 times of the patients with basal fractures and intracapsular fractures. When the mandibular body, mandibular angle were exerted external force from the side, it can cause flexural stress converging at the weakest part of condyle neck and excessive bending, which can easily lead to neck transverse fracture.

**Fig 10:**
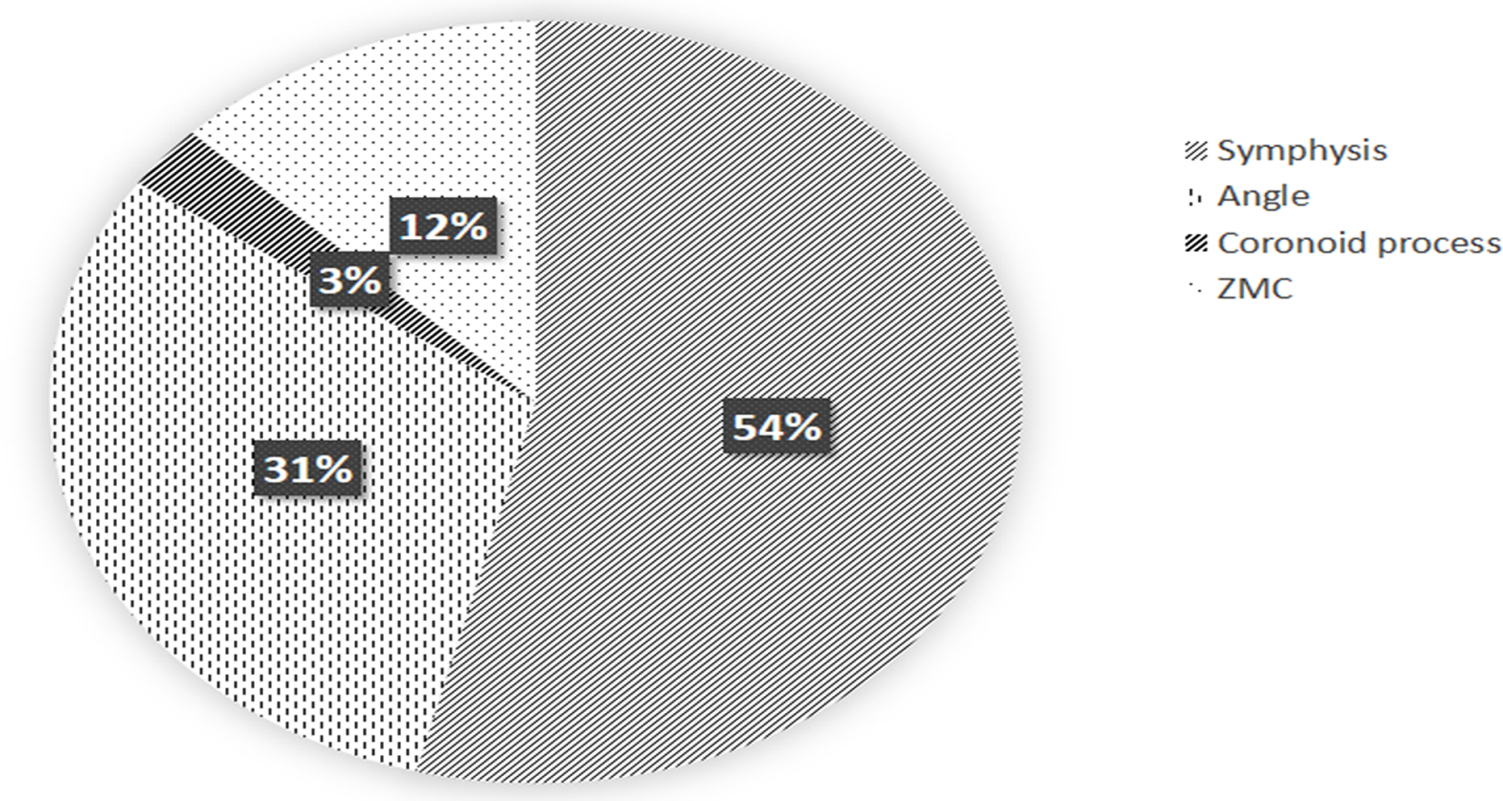
Statistical analysis of complex fracture involving damage to adjacent structures.

Most of the patients had combined injuries, accounting for 52.2% of chin injury, followed by 29.8% of mandibular angle injury, 7.9% of malar and zygomatic arch injury, 4.1% of orbital part injury, and 2.7% of coracoid process injury (Fig 10). This is consistent with the fact that chin is the most common part to be hit in accidents, falls and bumps, so that combined.

### The establishment of digital workflow for evaluation, diagnosis and treatment of condylar fractures

#### The applications of image segmentation, registration and 3D measurement in condylar fractures

Statistical analysis shows that there is no significant differences between 2D and 3D measurement (t = 1.65, P = 0.10). The 3D measurement chose a fixed mark point of the measurement system. So the data is stable, accurate, and intuitive. It can accurately measure the condylar fracture data, with high repeatability and stability[9, 10]. The combination of image registration and 3D measurement can shorten the measurement time for condylar fractures, which makes it highly feasible for condylar fracture research.

#### 180 cases with unilateral condyle fractures were analyzed in this study

Results: As shown in Tables 1–4, respectively.

**Table 1:** The relationship between PI-NRS and measurement index.

Pain index	Condylar Angle in coronal view(°)	Condylar diameter in coronal view(mm)	Ramus height(mm)	Condylar Angle in sagittal view(°)	Condylar neck Angle in sagittal view(°)
2	6.47	2.63	1.44	0.73	1.22
4	8.14	3.38	2.82	0.97	2.31
6	8.29	3.06	3.3	0.88	5.05
8	10.03	2.59	4.11	1.52	4.69
10	11.5	3.2	4.19	1.84	5.02

**Table 2:** The relationship between opening degree and measurement index.

Opening degree	Condylar Angle in coronal view(°)	Condylar diameter in coronal view(mm)	Ramus height(mm)	Condylar Angle in sagittal view(°)	Condylar neck Angle in sagittal view(°)
10mm	13.96	2.39	4.88	1.96	4.24
20mm	11.29	2.95	3.07	1.63	1.59
30mm	4.07	2.73	1.61	1.19	2.64

**Table 3:** The relationship between condylar angle changes and treatment effect.

Condylar Angle in coronal view(°)	Open reduction						Closed reduction					
	intracapsular fracture		condylar neck fracture		condylar fracture of the base		intracapsular fracture		condylar neck fracture		condylar fracture of the base	
	F	C	F	C	F	C	F	C	F	C	F	C
≥11°	15	13	58	17	45	22	13	14	10	24	6	15
<11°	6	5	10	8	6	7	5	6	10	12	5	7

(F:Free of disease, Patients recovered well without complications; C:Complications such as mouth opening restriction or ankylosis were found in patients.)

**Table 4:** The relationship between Ramus height changes and treatment effect.

Ramus height(mm)	Open reduction						Closed reduction					
	intracapsular fracture		condylar neck fracture		condylar fracture of the base		intracapsular fracture		condylar neck fracture		condylar fracture of the base	
	F	C	F	C	F	C	F	C	F	C	F	C
≥4mm	12	9	60	22	40	30	9	14	15	27	8	17
<4mm	4	3	6	9	4	4	4	6	12	15	3	6

(F:Free of disease, Patients recovered well without complications; C:Complications such as mouth opening restriction or ankylosis were found in patients.)

#### The establishment of standard process for digital diagnosis and treatment of condylar fractures

**Fig 11:**
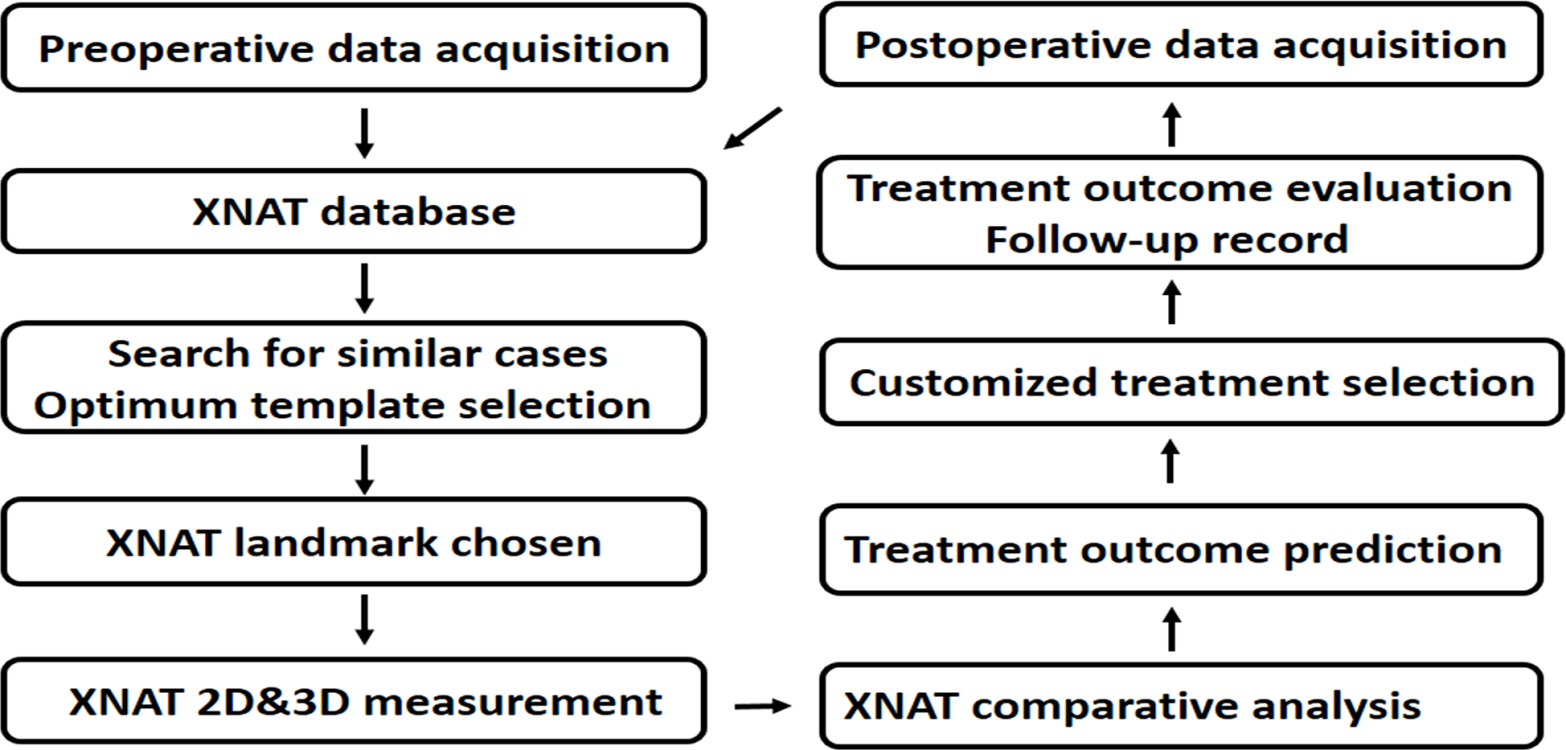
Overview of the digital workflow for evaluation, diagnosis and treatment of condylar fractures.

The diagnosis and treatment process of condylar fractures is shown in Fig 11.

After condylar fracture patients seeing doctors, their information entered the database, and their clinical data, image data, and follow-up treatments are stored in the database. After the patients are admitted to hospital, their image data are acquired, and measured by 3D measurement. According to the measurement data, digital diagnosis and classification are conducted, and a personalized treatment plan is developed. After the computer-assisted therapy finished, we will regularly follow up the prognosis of the patients, and input the data into the database, in order to facilitate the future data adjustment and optimize the digital diagnosis and treatment process for other patients.

## Discussion

### The application of 3D measurement and image registration in condylar fractures

The treatment of condylar fractures has long been controversial. Although the diseases exclusively for surgery or conservative treatment have been generally agreed, the individual treatment plan for complex patients is still a problem[11]. Digital technologies such as image segmentation, registration and fusion are helpful to 3D analysis on condyle fractures, and among them, 3D CT scan is necessary [12]. The use of digital technologies can help to achieve accurate measurement of the condyle shapes, comparative analysis of 3D morphological changes of condylar fractures before and after treatment, and dynamic detection of structural changes of fracture reconstruction at different stages, in order to establish the digital diagnosis and treatment standards for condylar fractures[13, 14].

### The treatment of condylar fractures

In this study, for the patients less than 18 years old without significant dislocation, surgery and conservative treatment were equivalent for intracapsular fractures, and had no difference for neck fracture and basal fracture. For the patients above 18 years old, there was no significant difference between the two methods for intracapsular fracture, but surgery treatment was slightly better than conservative treatment for condylar neck fracture and basal fracture.

The greater the condylar fracture shift angel was, and the more the mandibular ramus height reduction was, the stronger the pain they felt, and the more difficult for the patients to stand. When the pain reached 10 degrees, the condylar fracture angle was greater than 11 degrees, and mandibular ramus height reduction was greater than 4mm, the patient’s normal life was seriously affected. The greater the condylar fracture shift angle was, and the more the mandibular ramus reduction height was, the more difficult it is to open the mouth. When the patient’s mouth opening degree was only one finger, the patient's life quality would be severely affected. According to the above data, the mandibular ramus height and condyle angle have a greater impact on the surgery outcome.

In all 339 cases with condylar fractures, there are 252 cases with condylar fracture shift angle ≥11°, accounting for 74.3%, and 87 cases were with condylar fracture shift angle <11°, accounting for 25.7%. The condylar fracture shift angel has become one of the characteristics for condylar fractures. The larger the shift angle is, the greater the injury is, and the patients are more urgent for timely treatment.

When the condylar fracture shift angle was ≥ 11°, 170 cases received surgical treatment, including 118 cases with good mouth opening and face shape recovery, accounting for 69.4%, and 52 cases with complications like limited mouth opening or ankyloses, accounting for 30.6%; 82 cases received conservative treatment, with 29 cases (35.4%) having good prognosis and 53 cases (64.6%) having above complications. When the condylar fracture shift angle was <11°, 42 cases and 45 cases received surgical treatment and conservative treatment, with 22 and 20 cases having good mouth opening and face shape recovery, accounting for 52.4% and 44.4%, respectively.

After comparison, we found that in the 339 cases we analyzed, when the condylar fracture shift angel was ≥ 11°, the ratio of patients received surgical treatment and conservative treatment was 170: 82, and the ratio of patients with good recovery was 118: 29. Therefore, when the condylar fracture shift angel was ≥ 11°, the ratio of success rate for surgical treatment and conservative treatment was 4.1: 1. When the condylar fracture shift angel was < 11°, the ratio of patients received surgical treatment and conservative treatment was 1: 1, and the success rate was also similar, about 1.2: 1. This suggests that when the condylar fracture shift angel is ≥ 11°, surgical treatment is better than conservative treatment; when the condylar shift angle is <11°, these two treatment methods are equivalent.

In all 339 cases with condylar fractures, 263 cases had mandibular ramus height reduction ≥ 4mm, accounting for 77.6%; and 76 cases had mandibular ramus height reduction < 4mm, accounting for 22.4%. The mandibular ramus height reduction is also one of the characteristics for condylar fractures. The more the mandibular ramus height reduction is, the greater the injury is, and the patients are more urgent for timely treatment.

When the mandibular ramus height reduction was ≥ 4 mm, 173 cases received surgery, with 112 cases having good mouth opening and face shape recovery, accounting for 64.7%, and 61 cases having complications, accounting for 35.3%; 90 cases received conservative treatment, with 32 cases having good prognosis, accounting for 35.6%, 58 cases having complications, accounting for 64.4%. When the mandibular ramus height reduction was < 4mm, 30 and 46 cases received surgical treatment and conservative treatment, with 14 and 19 cases having good mouth opening and face shape recovery, accounting for 46.7% and 41.3% respectively.

After comparison, we found that in the 339 cases we analyzed, when the mandibular ramus height reduction was ≥ 4mm, the ratio of patients received surgical treatment and conservative treatment was 263: 76, nearly 3.5 times, and the ratio of patients having good recovery was 112:32, meaning that when mandibular ramus height reduction was ≥ 4mm, the ratio of success rate for surgical treatment and conservative treatment was 3.5: 1. When the mandibular ramus height reduction was < 4 mm, the patients received surgical treatment and conservative treatment was 1: 1, and the success rate of the two treatments was also similar, about 1.1: 1. Therefore, when the mandibular ramus height reduction was ≥ 4 mm, surgical treatment is better than conservative treatment; when the mandibular ramus height reduction was < 4 mm, these two treatments were equivalent.

## Conclusion

For the patients less than 18 years old, with no significant dislocation, surgical treatment and conservative treatment are equivalent for intracapsular fractures, and had no obvious difference for neck fractures and basal fractures. For the patients above 18 years old, there is no significant difference between the two methods for intracapsular fractures; but surgical treatment is slightly better than conservative treatment for condylar neck fractures and basal fractures. When condylar fracture shift angle is greater than 11 degrees, and the mandibular ramus height reduction is greater than 4mm, the patient feels the strongest pain, with mouth opening severely restricted and life quality greatly reduced. Such patients should receive surgical treatment. The digital evaluation system can objectively reflect the status of condylar fractures, and provide the basis for treatment. The establishment of XNAT condylar fracture database and the development of digital diagnosis and treatment process can provide basis for the future work such as digital telemedicine.

## Supporting information

S1 Table (PDF)
