# Supplementary material for: Digital diagnosis and treatment of mandibular condylar fractures based on Extensible Neuro imaging Archive Toolkit (XNAT)
Source: PLoS One. 2018 Feb 12;13(2):e0192831. doi: 10.1371/journal.pone.0192831 (PMC5809048; doi:10.1371/journal.pone.0192831)
Supplement: S1 Table — (PDF) [file pone.0192831.s001.pdf]

	Traditional 2D Measurement				
	Coronal view			Sagittal view	
ID	Ramus height(mm)	Condylar Angle(°)	Condylar diameter(mm)	Condylar diameter(mm)	Condylar Angle(°)
1	67.61	78.25	19.54	9.67	88.71
2	74.54	87.62	25.22	10.74	95.17
3	71.55	78.52	21.48	11.56	66.55
4	62.77	96.82	27.39	17.96	100.48
5	73.63	76.24	23.18	6.37	95.05
6	72.46	78.91	25.01	8.25	96.18
7	65.42	95.33	28	18.26	97.99
8	76.61	77.39	25.84	6.25	94.72
9	69.35	88.64	27.47	11.74	97.03
10	66.35	96.03	27	17.39	98
11	77.51	76.93	25.3	5.35	95.02
12	69.83	93.46	26.81	14.44	97.25
13	65.01	94.87	26.59	16.88	97.36
14	75.83	77.03	24.49	6.81	94.85
15	68.03	89.74	26.44	15.35	96.25
16	28.56	65.56	14.56	16.8	90.02
17	17.82	88.92	17.86	12.64	73.38
18	28.88	65.18	15.56	18.01	89.96
19	55.76	73.15	20.25	10.04	95.33
20	40.22	90.61	25.37	16.24	96.18
21	55.27	75.93	23.06	12.74	106.05
22	73.26	83.57	26.73	8.85	95.93
23	71.88	86.4	27.48	9.26	96.17
24	71.4	85.39	27.16	9.44	96.08
25	70.29	87.13	26.69	11.27	96.72
26	70.51	88.6	27.3	10.84	97.04
27	69.82	91.06	26.49	10.61	97.16
28	71.63	88.39	27.05	8.94	96.83
29	73.09	81.17	26.83	8.98	96.39
30	23.36	86.27	21.15	11.83	94.13
31	35.28	66.18	14.61	15.7	90.69
32	38.17	67.35	16.82	17.94	91.48
33	55.63	70.11	18.3	17.29	93.14
34	30.92	88.04	20.74	13.05	95.46
35	43.57	64.01	18.39	17.51	95.33
36	58.36	68.18	17.69	15.08	96.02
37	60.17	69.02	15.26	15.03	96.45
38	44.16	92.61	25.07	17.15	88.01
39	54.92	76.39	24.6	13.47	95.5
40	57.06	76.62	23.89	14.29	94.82
41	59.75	77.41	23.74	14.83	96.08
42	46.03	91.48	26.3	18.36	90.18
43	56.36	75.1	23.05	13.24	94.58
44	62.4	75.39	23.81	13.55	94.27
45	66.09	74.99	21.36	11.58	95.83
46	60.15	94.72	26.83	18.96	99.38
47	19.18	83.64	26.75	15.7	98.62
48	70.46	79.91	26.49	13.08	97.18
49	70.92	78.25	26.1	10.52	91.01
50	61.27	95.03	27.11	17.44	98.42

51	72.63	80.37	25.33	9.74	97.24
52	72.84	78.62	25.58	8.15	96.88
53	73.16	77.2	25	8.36	96.37
	3D Measurement				
	Coronal view			Sagittal view	
ID	Ramus height(mm)	Condylar Angle(°)	Condylar diameter(mm)	Condylar diameter(mm)	Condylar Angle(°)
1	67.82	78.43	18.99	9.15	88.64
2	74.76	87.14	25.2	9.22	95.26
3	70.98	79.23	21.44	11.26	66.25
4	62.8	97.02	27.52	17.74	99.92
5	74.21	76.22	23.24	6.35	94.89
6	72.56	78.58	25.45	8.21	96.21
7	65.38	96	27.69	18.25	97.53
8	76.83	77.31	25.84	6.3	94.84
9	69.33	88.67	27.48	11.75	97.1
10	66.35	96.12	27.38	17.25	98.12
11	77.65	76.87	24.98	5.63	94.86
12	70.12	93.23	27.55	13.96	97.25
13	65.37	94.85	26.54	14.5	97.32
14	76.12	77.16	24.35	16.84	97.36
15	67.96	90	26.82	15.42	96.32
16	28.56	65.68	14.98	16.79	90.23
17	17.83	88.96	17.46	12.65	73.38
18	28.39	65.23	15.36	18.05	89.77
19	56.01	73.12	20.22	10.13	96.34
20	40.22	90.65	25.36	15.98	96.32
21	55.22	76.21	23.15	12.74	105.96
22	73.26	83.52	26.73	8.86	95.94
23	71.86	86.45	27.45	9.23	96.52
24	71.23	85.36	27.14	9.44	96.28
25	70.35	87.13	26.67	11.27	96.75
26	70.56	88.59	27.3	10.85	97.21
27	69.86	91.05	26.64	10.63	97.58
28	71.26	88.36	27.05	8.99	96.91
29	79.09	81.15	26.54	8.95	96.46
30	23.34	86.28	21.32	11.83	94.25
31	35.29	66.35	14.51	15.75	90.65
32	38.32	67.49	16.88	17.94	91.48
33	54.85	69.29	18.35	17.28	93.13
34	30.89	88.1	20.69	13.11	95.48
35	43.52	64.14	18.54	17.56	95.41
36	58.36	68.23	17.68	15.1	95.99
37	60.2	69.11	15.29	15.03	96.44
38	44.23	92.65	25.11	17.15	88.2
39	54.89	76.66	24.58	13.38	95.51
40	57.12	76.66	28.69	14.34	94.85
41	60	77.41	23.78	14.82	96.05
42	46.12	91.51	26.28	18.31	90.15
43	56.36	75.11	22.98	13.22	94.58
44	62.44	75.38	23.79	13.55	94.25
45	66.11	74.97	21.33	11.74	95.82
46	60.15	94.8	26.83	18.89	99.36
47	19.2	83.66	26.75	15.69	98.64

48	70.45	79.93	26.46	13.07	97.21
49	70.99	78.26	26.03	10.55	91.1
50	61.25	95.02	27.11	17.29	98.47
51	72.57	80.33	25.35	9.77	97.25
52	72.88	78.62	25.57	8.36	97.01
53	73.16	77.19	25.31	8.39	96.34
